# Effect of fish oil omega-3 fatty acids on reduction of depressive symptoms among HIV-seropositive pregnant women: a randomized, double-blind controlled trial

**DOI:** 10.1186/s12991-018-0220-4

**Published:** 2018-11-26

**Authors:** Rose Okoyo Opiyo, Peter Suwirakwenda Nyasulu, Reuben Kamau Koigi, Anne Obondo, Dorington Ogoyi, Wambui Kogi-Makau

**Affiliations:** 1 School of Public Health, University of Nairobi, P.O. Box 30197, Nairobi, 00100 Kenya; 2 Division of Epidemiology & Biostatistics, Faculty of Medicine and Health Sciences, Stellenbosch University, Cape Town, South Africa; 3 Department of Obstetrics and Gynecology, University of Nairobi, P.O. Box 30197, Nairobi, 00100 Kenya; 4 Department of Psychiatry, University of Nairobi, P.O. Box 30197, Nairobi, 00100 Kenya; 5 P.O.Box 52428, Nairobi, 00200 Kenya; 6 Department of Food Science, Nutrition and Technology, University of Nairobi, P.O. Box 30197, Nairobi, 00100 Kenya

**Keywords:** Omega-3, Fish oil, Depression, HIV infection, Pregnancy, RCT

## Abstract

**Background:**

Globally, it is known that HIV-infected pregnant women are prone to depressive symptoms. Research evidences also suggest that nutrient deficiencies may enhance the depressive illness, and that fish oil omega-3 fatty acids may alleviate the depressive symptoms. The aim of this study was to assess the effect of fish oil omega-3 eicosapentaenoic acid-rich supplements on depressive symptoms among HIV-seropositive pregnant women.

**Trial design:**

A randomized double-blinded controlled trial with two parallel groups was conducted. The intervention group received fish oil omega-3 of 3.17 g (eicosapentaenoic acid = 2.15 g; docosahexaenoic acid = 1.02 g) per day for 8 weeks, while the control group received soybean oil for a similar period.

**Method:**

Participants were HIV-seropositive pregnant women who were enrolled in prevention of mother-to-child transmission programs and attending antenatal clinics at selected Nairobi city county’s health facilities. Recruitment was done from health records of HIV-infected pregnant women. Data analysis followed per-protocol analysis. Participants who completed the 8-week trial were included in the analysis of covariance statistical model with omega-3 as main effect. The covariates in the change in BDI-II depressive symptom score outcome were baseline characteristics and nutrient adequacy.

**Results:**

282 participants were recruited 109 randomized to fish oil, and 107 to soybean oil. Completion rate was 86/109 (78.9%) and 96/107 (89.7%) respectively. At the end of week-8 of follow up most participants in both arms had mild depressive symptoms 82/86 (95.3%) in the Fish oil group and 94/96 (97.9%) in the Soybean oil group. The difference in effect between the intervention and control group was not statistically significant (1.01 (95% CI − 0.58 to 2.60), *p *= 0.21).

**Conclusion:**

Fish oil omega-3 with a daily dosage of 3.17 g (eicosapentaenoic acid = 2.15 g; docosahexaenoic acid = 1.02 g) appears to provide no added benefit in reduction of the symptoms of depression in HIV-infected pregnant women.

*Trial Registration* Clinical Trial Registry: NCT01614249. Registered on June 5, 2012. https://clinicaltrials.gov/ct2/show/NCT01614249

## Introduction

During pregnancy, women may experience at least one episode of minor or major depression referred to as perinatal or maternal depression. The depression in pregnant women is, however, often under-diagnosed, undetected and missed out due to lack of routine screening [1]. Research findings on depression in pregnancy indicate that 20–40% of pregnant women are depressed [2–4]. Globally, it is also known that human immunodeficiency virus (HIV)-infected pregnant women are prone to depression [5–8]. The depression symptoms in HIV-infected pregnant women may be due to the effects of pregnancy, HIV infection or a combination of both pregnancy and HIV infection. Some symptoms of depression such as fatigue, mood disorders, changes in appetite, or changes in sleeping pattern have been known to overlap with the symptoms present in pregnancy, HIV infection or due to side effects of HIV medication [9]. Among HIV-positive pregnant women in Sub-Saharan Africa, Smith Fawzi, Kaaya [10] reported a prevalence of elevated depressive symptoms of 42.4% in Tanzania. Manikkam and Burns [11] also established a depression prevalence of 38.5% among HIV-infected pregnant women in KwaZulu-Natal, while Rochat, Richter [12] reported a prevalence of 47% from rural South Africa and Kwalombota [13] reported a high prevalence of major depression of 85% from Zambia. These research evidences suggest that depression in HIV-infected pregnant women is a public health problem.

Evidences suggest that nutrient deficiencies may enhance the depressive illness and in pregnancy and HIV infection, nutrients’ demand is higher than normal due to increased need by the developing fetus in the context of nutrient deficiencies that result from reduced food intake, impaired nutrient absorption and altered metabolism due to the HIV status [14]. Yet, inadequate and inappropriate dietary intake has been reported globally among pregnant women. For example, in the United States of America [15], Canada [16], Europe [17, 18], Australia [19], India [20] and Africa [21], studies indicate that micronutrients and omega-3 fatty acid intake values are below the recommended levels for normal healthy life. It is also globally known that fish oil omega-3 fatty acids may alleviate the depressive symptoms in pregnancy [22–25]. Inconsistencies exist in several previous studies [25–28]. These variations could be attributed to differences in the ratio of omega-3 long-chain fatty acids of eicosapentaenoic acid (EPA) and docosahexaenoic acid (DHA), depression assessment tools, intervention duration or physiological status of study participants, which varies by study. Although omega-3 DHA is recognized as an essential structural component of the cell membranes in the central nervous system [29], some evidence suggest that omega-3 EPA may be more beneficial in reduction of depression than DHA, based on its anti-inflammatory effects of the metabolic products of eicosanoids and its oxidized derivatives as well as its efficacy at reducing the inflammatory cytokines [30, 31]. These metabolic products of EPA are important in controlling the cellular inflammation in the CNS that may manifest as depressive symptoms. A higher proportion of omega-3 EPA than DHA in the fish oil omega-3 supplement at a higher ratio has, therefore, been presumed to be more beneficial in reduction of depressive symptoms [31, 32]. This is demonstrated in the study by Freeman and colleagues [22] who used a lower omega-3 fatty acid ratio of 1.1 g/day EPA and 0.8 g/day DHA and found no significant benefit of omega-3 over placebo, and, Su and colleagues [25] who reported a significant reduction in depression with higher omega-3 content of 2.2 g/day EPA and 1.2 g/day DHA.

There is paucity of published research on the effect of fish oil omega-3 on depression among the HIV-infected pregnant women. Yet, while depression is common in pregnancy, HIV infection is also a factor that induces depression, a combination of the two may cause severe depression effects hitherto unaddressed. In HIV infection, opportunistic infections in the central nervous system may affect the patients cognitive, motor and behavioural functioning, and, manifest as psychiatric disturbances including depression [33]. The depression in HIV-infected pregnant women may also negatively impact on the progression of HIV disease [5, 8] and may adversely affect the quality of life and adherence to HIV/AIDS medication regimens [10, 34, 35]. The studies that have been conducted on omega-3 fatty acids in HIV infection have focused on the effect on triglycerides and cluster of differentiation 4 (CD4) cell count. For example, omega-3 fatty acid supplementation has been shown to reduce triglyceride levels in patients receiving antiretroviral [36, 37]. Omega-3 fatty acid supplementation has also been shown to increase CD4 cell count as demonstrated [38, 39]. To our knowledge, therefore, no previous studies had been undertaken on the effect of fish oil omega-3 fatty acids on depressive symptoms among HIV-infected pregnant women.

The research hypothesis for our study was derived from the evidence that omega-3 can alleviate symptoms of depression [25, 26, 28, 40–42]. If omega-3 EPA fatty acid is the most important compound in alleviating depressive symptoms [25, 26, 28], then depressed individuals taking fish oil omega-3 supplements should experience a change in severity of their depressive symptom condition. A 50.0% reduction in depressive symptom scores after 8 weeks of supplementation with fish oil (EPA = 2.2 g/day; DHA = 1.2 g/day) had been earlier reported [25]. Sources of omega-3 fatty acids have also influenced the designing of studies on fish oil omega-3 and depression. Whereas fish oil contains long-chain omega-3 EPA and DHA fatty acids, plant-based edible oils like soybean contain short-chain omega-3 ALA which must be metabolized after consumption to EPA and DHA in the body, hence the choice of soybean oil for the control group. Most clinical trials on long-chain omega-3 EPA and DHA fatty acids have also used plant-based oils such as olive oil, soybean, canola, palm oil, corn oil or sunflower [43] for the control group in fish oil omega-3 interventions.

## Methods

### Participants

The study population was HIV-positive pregnant women enrolled in the Prevention of Mother to Child Transmission (PMTCT) of HIV/AIDS programs and attending antenatal clinics (ANC) at Nairobi City County’s health facilities of Riruta, Mathare North, Kariobangi North and Kayole-II Sub-district Hospital. All HIV-seropositive pregnant women were eligible to participate in the study if they had known CD4 cell count of not more than 500 cells/µl, were on antiretroviral therapy (ART) and were in their second trimester of pregnancy at 14–27 weeks. Only those who consented to participate by signing the consent form and had at least 14 scores on Beck Depression Inventory Second Edition (BDI-II) scoring questionnaire were enrolled.

Participants were excluded from the study if they were underweight with a mid-upper arm circumference (MUAC) less than 22 cm and overweight with MUAC of more than 33 cm. They were also excluded if they had a medical history of use of a blood thinning medication/anti-clotting medication for health conditions like liver problem, varicose veins, peptic ulcers; or use of Vitamin K supplement since omega-3 may increase the effects of these medications. Those who had used antidepressant medications 2 weeks prior to the study or were on diabetic medication to lower blood sugar were also excluded from the study.

Ethical approval to conduct the study was obtained from the Kenyatta National Hospital/University of Nairobi Ethical Review Committee (KNH/UoN-ERC, P266//6/2011), the Ministry of Education, Research and Technology (NCST/RRI/12/1/MED011/167) and from the Pharmacy and Poisons Board of Kenya (PHD/MOH/R1/101/2011/ac). Furthermore, only those who agreed to participate by signing the consent forms after receiving the relevant information about the study from the research team were enrolled in the study.

### Interventions

The intervention group received three soft gels of fish oil omega-3 fatty acid for each day, each containing more EPA (0.715 grams) than DHA (0.340 grams). For the intervention group, therefore, the total daily intake of omega-3 fatty acids from the three fish oil soft gels was 3.17 grams (EPA = 2.15 grams per day; DHA = 1.02 grams per day) for each participant. The control group received three soft gels of soybean oil per day, each containing saturated fatty acids (0.178 grams), monounsaturated fatty acids (0.299 grams) and polyunsaturated fatty acids (0.985 grams) with traces of EPA (0.115 grams). Both the fish oil omega-3 supplements and soybean oil soft gels were provided by *Innovix Pharma Inc, California*, manufacturers of *OmegaVia* fish oil products. These products were both similar in shape, color and taste.

Participants carried the fish oil omega-3 and soybean oil soft gels home to take orally, one three times per day: in the morning, mid-day and in the evening after meals for a period of 8 weeks. The participants received the trial product supplies to last of them for 2 weeks, after which they came to the sites for follow-up visits for face-to-face monitoring of side effects and compliance as well as re-supply of the products. Each participant was encouraged to use the research cell-phone contact on the container for the products to contact the research team whenever necessary. Regular cell-phone contacts were established with each participant immediately after enrolment in the study. Participants were also contacted on the cell-phone details they provided during recruitment at least two times a week to monitor intake of the soft gels and to remind them of the next visit to the study site. Further confirmation as to whether all the soft gels were swallowed was done during the bi-weekly face-to-face visits for re-supply of trial products and data collection. During these visits, each participant was asked whether she had any soft gel left in her container at home, and the number. Participants were expected to have remained with only one or two soft gels in their last container before starting the next re-supply container. Any participant, who reported more soft gels left in her container than the expected number, was required to give reasons for non-completion. Participants who had soft gels for 5 days or more were dropped out of the study for non-compliance. Those who were non-compliant and had depressive symptoms were referred to the doctor in the same health facilities for medical attention and counseling.

### Objective and hypothesis

The objective of this study was to determine the effect of fish oil omega-3 EPA-rich supplements on BDI-II depressive symptom scores among HIV-seropositive pregnant women.

We hypothesized that there is a difference in the magnitude of change in depressive symptom scores of at least 20% as measured by BDI-II scale among HIV-seropositive pregnant women with depressive symptoms taking fish oil omega-3 EPA-rich supplements than the control group taking soybean oil soft gels whose alphalinoleic acid are minimally metabolized to EPA [44, 45]. The null hypothesis of this study was that there is no difference in the magnitude of change in BDI-II scores between HIV-seropositive pregnant women with depressive symptoms taking fish oil omega-3 EPA-rich supplements and the control group taking soybean oil soft gels.

### Outcomes

The primary outcome variable of the study was the change in depressive symptom scores between baseline scores and the scores after 8 weeks as measured by BDI-II scale. The Beck Depression Inventory Second Edition (BDI-II) Scale is a 21-item scoring tool which measures the existence and severity of symptoms of depression. Each of the 21 items on BDI-II tool represents a depressive symptom. The symptoms are each scored on a 4-point Likert scale of 0–3 (0 = symptom is absent; 3 = symptom is severe). Scores for each symptom was added up to obtain the total scores for all 21 items, which are interpreted as follows: Scores of 0–13: minimal depression; 14–19: mild depression; 20–28: moderate depression and 29–63: severe depression.

This outcome is similar to what Su and colleagues used in their study from Taiwan [25]. The quality of this outcome variable was enhanced in our study in several ways. First, the intervention allocation ratio of 1:1 used ensured a balance in allocation and minimization of within-group variability. Second, the participants and those administering the trial, including the principal investigator were blinded to the trial allocation. Third, standard operating procedures were developed and used as step-by-step guidelines to ensure uniformity in intervention implementation and data collection as per the research protocol. Fourth, the research team was trained on data collection tools and principles of good clinical practices (GCP). They undertook the GCP online course at East African Consortium for Clinical Research (EACCR) website http://www.eaccr.org/nodes/training/. Fifth, a Data and Safety Monitoring Board (DSMB) was constituted to monitor the safety and treatment efficacy of the omega-3 supplements during the clinical trial period. The DSMB was an independent group of experts comprising of a pharmacist, gynecologist, biostatistician and a psychiatrist. Sixth, dietary intake data for locally available omega-3 rich foods were collected and controlled for as a potential confounder in the statistical analysis. Seventh, participants were regularly monitored for compliance in taking the trial products.

### Sample size

A sample size of 91 women per study group gave an 85% power to detect as statistically significant at 5% level (*α* = 0.05), a true difference of four scores in the mean depressive symptom scores between women given fish oil omega-3 EPA-rich supplements and women given soybean oil, assuming a within-group standard deviation of nine in depressive symptom scores. Assuming that the mean depressive symptom scores after 8 weeks in the control group was 20 scores; a decrease of four units corresponded to a 20% change in the depressive symptom scores.

The sample size was determined based on an assumption of omega-3 intervention difference of lowering depressive symptom scores by an estimated 20% as measured by Beck Depression Inventory, Second Edition (BDI-II) Scores. Su and colleagues reported a 50% reduction in depressive symptom scores after 8 weeks of intervention with fish oil omega-3 [25]. A change of 20% would be a change of at least 4 units. The sample size was adjusted for a 10% non-response due to drop-outs and losses to follow-ups; the following formula was used to calculate the final adjusted sample size: n* = *n*/(1 − *q*), where n* = adjusted sample size, *n* = sample size before adjusting, and *q* = the proportion expected for non-response [46], estimated at 10%. When adjusted for the 10% non-response due to drop-outs, a total of 200 participants with at least mild depressive symptoms [47] were enrolled in the study, with 100 participants randomized in each of the study groups. However, during the trial, 16% of the study participants dropped out instead of the planned 10%. An additional 16 participants were, therefore, recruited with approval from Kenyatta National Hospital/University of Nairobi Ethical Review Committee (KNH/UoN-ERC), the institutional review board. Hence, a total of 216 participants were enrolled in the study, 109 on fish oil omega-3 intervention and 107 on soybean soft gel control arm.

### Randomization procedure

Randomization allocation sequence was computer generated. Block randomization of four fixed blocks was used, with a 1:1 allocation ratio used to achieve balance in allocating the intervention to participants in the four study sites and to reduce within-group variability. For every block of four participants, two were allocated to each intervention group. The four blocks used were consistent with the four study sites of Kariobangi, Riruta, Mathare and Kayole health centers. Six possible balanced combinations of assignment of either fish oil or soybean oil group in four blocks were computer generated as: BBAA BABA, BAAB, ABBA, ABAB and AABB. Participants were allocated to each group by randomly selecting one of the six combinations and assigning them an identification code according to the specified sequence as described by Efird [48].

### Randomization allocation concealment

Allocation concealment refers to the technique used to implement the randomization sequence [49] by protecting the randomization list and allocation codes such that the allocated intervention is not known until the study is un-blinded. We achieved the randomization allocation concealment in this study by involving someone who did not participate in recruitment, enrolment, data collection and monitoring or data management to allocate the intervention to participants using plastic bottles. The plastic bottles were identical, opaque, securely sealed and sequentially numbered according to the study randomization sequence generated. The randomization sequence was concealed by the independent statistician until the interventions were assigned. Hence, only the independent statistician who generated the randomization sequence and the person who allocated the intervention were not blinded to the study.

### Randomization implementation

The randomization allocation sequence was generated by an independent statistician who was not involved in the study to ensure allocation concealment of the intervention. Participants were recruited in the study by mentor mothers of mother2mothers (m2m) non-governmental organization who provided peer education and psychosocial support in PMTCT. They were assigned to either of the trial arms by the data collection team soon after enrollment and completion of the study questionnaires to minimize dropouts, which might have occurred if enrolled participants exceeded gestation age or gave birth before randomization.

### Blinding

This study was blinded to the participants and those administering the trial, including the principal investigator, were unaware of the intervention/treatment allocation. Specifically, the following individuals involved in the trial activities were blinded from the trial: principal investigator, study participants, health facility personnel, and research assistants and data clerk handling the data. Un-blinding of the trial products was performed after completion of data collection, data entry and preliminary analysis by the independent statistician who had generated the randomization sequence. The un-blinding was done in the presence of the researcher, and witnessed by at least one academic supervisor and the director of the school. This procedure of un-blinding the trial completely eliminated any source of bias during administration of the study products.

### Statistical methods

The primary outcome variable, change in BDI-II depressive symptom scores, was computed as suggested by Jamieson [50] in his article on analysis of covariance (ANCOVA) with difference scores as follows: *Post*-*intervention BDI*-*II scores (at week 4 or week 8) minus baseline BDI*-*II scores (at week 0).* The change in BDI-II scores at weeks four (mid-study) and eight (end of study) were tested for normality and summarized as mean values with standard deviations (SD), reporting 95% confidence interval levels (95% CI). The magnitude and difference in change in BDI-II depressive symptom scores were compared between the two study groups by Student *t* test. Before adjusting for baseline covariates, simple linear regression model was fitted to determine the variability in change in BDI-II scores that was explained by the intervention. The level of significance for all the inferential tests was at 5%. Regression to the mean effect by any suspected extreme values was controlled by randomization at the study design stage and by analysis of covariance (ANCOVA) model during data analysis. The ANCOVA adjusts each participant’s follow-up measurement according to their baseline measurement [51]. The analysis followed per-protocol analysis approach, where participants who did not complete the trial were not included for the primary outcome analysis at the end of the study. Those who dropped out of the study before the end of 4 weeks after enrolment were not included in the mid-study analysis. Similarly, all participants who dropped out of the study before the end of the trial at 8 weeks were not included in the final data analysis.

The intervention effect was compared between the two groups by fitting the ANCOVA regression model. The model included change in BDI-II scores after intervention, all the baseline characteristics and intervention arms. All baseline characteristics were included in the model as covariates to adjust for any possible variations at baseline before randomization into fish oil intervention group or soybean control group, and to control for regression to the mean. Bivariate and multiple linear regression analysis were used to determine which variables were key determinants of BDI-II depressive symptom scores. Heteroskedasticity due to differences in variance in the errors across observations was controlled by robust standard errors analysis in the regression model. The presence of interaction between the covariates (predictor variables) that were found to be significantly associated with the change in BDI-II scores in the ANCOVA model was tested. There was no interaction between the predictor variables.

## Results

### Participant flow and baseline characteristics

**Fig. 1:**
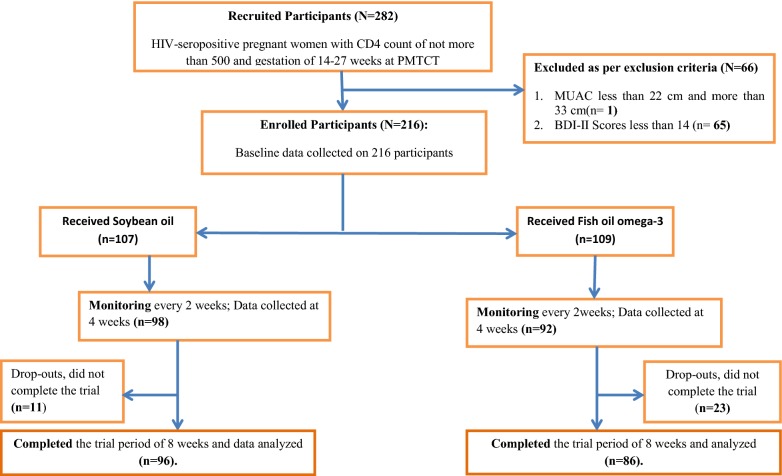
Participants’ distribution and flow during the trial based on the Consolidated Standards of Reporting Trials. The participants’ distribution and flow during the trial based on the Consolidated Standards of Reporting Trials [52]. It indicates total number of study participants who were recruited (*N* = 282), excluded from enrolment based on exclusion criteria (*N* = 66), enrolled for the 8-week study (fish oil arm = 109; soybean oil arm = 107), dropped out before the 8 weeks (fish oil arm = 23; soybean oil = 11) and those who completed the 8-week trial period and were included in the analysis (fish oil = 86; soybean oil = 96).The difference in completion between the two groups was, however, not significant at the end of study (X^2^(1) = 1.64; *p* value = 0.20), and did not significantly influence the change in BDI-II depressive symptom scores in fish oil (0.41 (95% CI − 4.49 to 5.32), *p *= 0.87) and soybean oil (− 3.21 (95% CI − 13.18 to 6.76), *p *= 0.52) groups

The participant flow throughout the study is as shown in Fig. 1. We recruited 282 pregnant women who met the inclusion criterion of HIV-seropositive with CD4 count of not more than 500 cells/mm^3^and gestation of 14 to 27 weeks of pregnancy. The study was conducted from July 2012 to August 2013. After screening for enrolment, 66 of the 282 were excluded due to exclusion criteria of mid-upper arm circumference (MUAC) measurement of more than 33 cm (*n* = 1) and depressive symptoms scores being less than 14 (*n* = 65). During the trial, 16.0% (34/216) of the participants dropped out instead of the planned 10%. An additional 16 participants were, therefore, recruited with approval from the institutional ethical review board, KNH/UoN-Ethical Review Committee. Hence, a total of 216 participants were enrolled and randomly assigned into the two study arms: 109 received fish oil omega-3 and 107 received soybean oil soft gels, each for 8 weeks. A total of 182 participants completed the 8-week study period: 86 from the omega-3 fish oil group and 96 from soybean oil group.

Overall completion rate was 84.3% (182/216) for all the enrolled participants. The completion rates were 78.9% (86/109) and 89.7% (96/107) for fish oil omega-3 and soybean oil arms, respectively. The difference in completion between the two groups was, however, not significant at the end of study (*X*^2^(1) = 1.64; *p* value = 0.20), and did not significantly influence the change in BDI-II depressive symptom scores in fish oil (0.41 (95% CI − 4.49 to 5.32), *p *= 0.87) and soybean oil (− 3.21 (95% CI − 13.18 to 6.76), *p *= 0.52) groups. A total of 34 of them dropped out; 23 from omega-3 arm and 11 from the soybean arm, respectively. Among the 34 participants who did not complete the 8-week study period, 21 of them had traveled to the rural homes, nine failed to return for bi-weekly re-supply for unknown reasons and four of them gave birth before the end of the trial.

### Characteristics of participants at baseline

#### Demographic and socio-economic and characteristics of participants

The participants’ characteristics of demographic, socio-economic, health and HIV status at baseline are shown in Table 1. The age distribution was similar in both groups with a median and interquartile range (median (IQR)) of 26 (22–30). The median (IQR) gestational age was 22 (18–24) and 22 (19–24) weeks for fish oil and soybean oil groups, respectively. The total household income per month in Kenya Shillings was a median of 6000 (3000–8000) for fish oil group and 5025 (3000–8000) for the soybean group. This was the income from the participant and her spouse, together with any other person living with them. Based on this income level, the proportion of households that were below the poverty line, earning less than Kenya Shillings 2913, were also similar in the two groups with 17.5% in fish oil omega-3 and 19.4% in soybean oil group.

**Table 1:** Participants’ baseline characteristics by study group

Baseline characteristic	Fish oil intervention group (*N *= 109)	Soybean oil control group (*N *= 107)
Continuous variables (median (IQR))		
Age (single years)	26 (22–30)	26 (22–30)
Gestation age (weeks)	22 (18–24)	22 (19–24)
CD4 count (cells/mm^3^)	361 (287– 440)	360 (288–414)
Weight (Kg)	60.5 (54.2–66.4)	62.0 (57.2–69.0)
MUAC (cm)	26.0 (23.9–27.6)	26.0 (24.3–28.0)
Household income per month (KSh)	6000 (3000–8000)	5025 (3000–8000)
BDI-II depressive symptom scores	20 (16–25)	21 (17–25)
Categorical variables (*n* (%))		
Age group (in years)		
Young = 15–25	53/109 (48.6)	48/107 (44.8)
Not young 26–45	56/109 (51.4)	59/107 (55.1)
Marital status		
Single (not married, divorced, widowed)	27/109 (24.8)	22/107 (20.6)
Married	82/109 (75.2)	85/107 (79.4)
Parity status		
First pregnancy	24/109 (22.0)	24/107 (22.4)
Not a first pregnancy	85/109 (78.0)	83/107 (77.6)
Education status		
No high school education	55/109 (50.4)	55/107 (51.4)
At least high school education	54/109 (49.5)	52/107 (48.6)
Employment status		
Not in gainful employment	61/109 (56.0)	64/107 (59.8)
In gainful employment	48/109 (44.0)	43/107 (40.2)
Household income status per month		
Below poverty line (less than Ksh.2913)	16/91 (17.5)	20/94 (19.4)
At least on poverty line (at least Ksh.2913)	75/91 (82.4)	74/94 (78.7)
Experienced stressful life events		
No stressful event 2 weeks before the study	73/109 (66.7)	75/107 (70.1)
Had stressful event 2 weeks before the study	36/109 (33.0)	32/107 (30.0)
CD4 cell count levels**		
Less than 350 cells/mm^3^	44/108 (40.7)	47/107 (43.9)
350–500 cells/mm^3^	64/108 (59.3)	60/107 (56.1)
Knew HIV status before pregnancy		
Newly tested (Less than 6 months)	56/109 (51.4)	56/107 (52.3)
Known positive (KP) 6 months or more	53/109 (48.6)	51 (47.6)
HIV status disclosure to anyone		
Status not disclosed to anyone	18/109 (16.5)	25/107 (23.4)
Status disclosed to someone	91/109 (83.5)	82/107 (76.6)
PMTCT support group meetings attendance		
Not attended support group meeting	62/109 (56.8)	54/107 (50.4)
Attended support group meeting	47/109 (43.1)	53/107 (49.5)

**CD4 cell count levels: Fish oil—*n *= 108 (1 participant with missing CD4 was lost to follow-up)

At least three-quarters of participants in each group were married and living with their spouse, and for more than 75% of them, this was not their first pregnancy (fish oil omega-3 = 78.7%; soybean = 78.3%,). It was also noted that participants had similar education status where those with at least high school education were 49.5% in fish oil group and 48.6% in soybean oil group. Experiences of stressful life events 2 weeks prior to the study were also similar in both intervention arms (33.0% in fish oil and 30.0% in soybean oil group). The stressors included knowledge of HIV status before pregnancy and disclosure (fish oil = 39.0%; soybean oil = 51.5%), domestic- and marital-related problems (fish oil = 25.0%; soybean = 45.0%) and financial-related issues (Fish oil = 36.1%; soybean oil = 3.0%).

#### Health-related characteristics at baseline

At baseline, participants in both groups had mild to moderate and severe depressive symptoms (Fish oil: mild = 47/109 (43.1%); moderate = 46/109 (42.2%); severe = 16/109 (14.7%); soybean oil: mild = 46/107 (43.0%); moderate = 48/107 (44.8%); severe = 13/107 (12.1%).The minimum BDI-II symptom score in both groups was 14, while the maximum was 40, and the median scores were 20 (IQR = 16–25) and 21 (IQR = 17–25) in fish oil intervention and soybean oil control groups, respectively. These baseline BDI-II scores were similar in the two study groups before randomization. The CD4 cell count (cells/mm^3^) level was also similar for participants in both intervention arms. In fish oil omega-3 group, it was (median (IQR)) 361 (287–440) and in soybean oil group it was 360 (288–414) (*p *= 0.39). About half of participants from each intervention group (Fish oil = 51.4% had knowledge of their HIV status at least 6 months before the pregnancy, while the other half had been newly tested for HIV. More than 75% of them from each group had disclosed their status to a friend, a relative or husband.

Although all HIV-infected pregnant women were routinely invited by m2m peer educators to attend support group meetings as part of the PMTCT program, only 47/109 (43.1%) and 53/107 (49.5%) of participants from fish oil and soybean oil reported to have attended these meetings. We observed that during these m2m support group meetings, the HIV-infected pregnant women received support and encouragement from the m2m peer educators and women who were either pregnant or had given birth with HIV infection. We also observed that these meetings involved sharing experiences of HIV-infection, pregnancy and childcare, followed by a health talk by m2 m peer educators. It was further observed that sharing a meal at the end of the m2m health talk gave participants an opportunity to interact with each other and share their own experiences or listen to others.

#### Participants’ dietary nutrient intake at baseline

The dietary nutrient intake values from the reported foods and the proportion of study population reporting consumption levels below the Estimated Average Requirements (EAR) for pregnant women are as listed in Table 2. At baseline, the median nutrient intake was below the EAR for pregnant women for all nutrients consumed by participants in each of the study groups. The intake values were least for omega-3 EPA and DHA fatty acids whose median (IQR) values were zero in the Fish oil group and 0 (0–0.01) grams in the Soybean oil group. More than 60.0% of participants from each intervention arm had their dietary nutrient intake levels below the EARs for pregnant women for most nutrients.

**Table 2:** Dietary nutrient intake levels reported at baseline

Nutrient	Estimated average requirements (EAR)	Baseline nutrient intake levels (median (IQR)) and proportions of participants with less than EAR^a^ levels for pregnant women			
		Fish oil arm		Soybean oil arm	
		Nutrient intake levels (Median (IQR))	Proportion with below EAR levels (n/arm total (%))	Nutrient intake levels (Median (IQR))	Proportion with below EAR levels (n/arm total (%))
Vitamin C (mg)	71	63.5 (15.0–121.6)	62/109 (56.9)	61.5 (15.1–191.2)	59/107 (55.1)
Vitamin B1 (mg)	1.2	0.8 (0.2–1.8)	73/109 (67.0)	0.7 (0.2–1.8)	67/107 (62.6)
Vitamin B6 (mg)	1.6	0.7 (0.3–1.4)	85/109 (78.0)	0.7 (0.4–1.3)	86/107 (80.4)
Vitamin B12 (mcg)	2.6	0.5 (0–1.4)	97/109 (89.0)	0.4 (0–1.4)	94/107 (87.8)
Folate (mcg)	520	106.4 (46.2–302.7)	98/109 (89.9)	178 (23.7–360.0)	91/107 (85.0)
Iron (mg)^b^	22.0	1.8 (0.6–3.6)	109/109 (100)	2.3 (1.1–3.2)	107/107 (100)
Vitamin E (IU)	12.5	4.6 (2.5–8.6)	89/109 (81.6)	4.7 (2.0–9.6)	87/107 (81.3)
Calcium (mg)	833	411.0 (238.1–916.1)	78/109 (71.2)	448.8 (206.5–930.6)	79/107 (73.8)

^a^EAR: Estimated Average Requirements were computed by dividing the recommended dietary allowances (RDAs) for pregnant women by the corresponding conversion factor for each nutrient

^b^Iron: Median dietary intake values for iron were computed at bioavailability levels of 15%

### Effect of fish oil omega-3 EPA-rich supplements on change in BDI-II depressive symptom scores among HIV-seropositive pregnant women

#### Normality tests for change in BDI-II scores by study arm

**Fig. 2:**
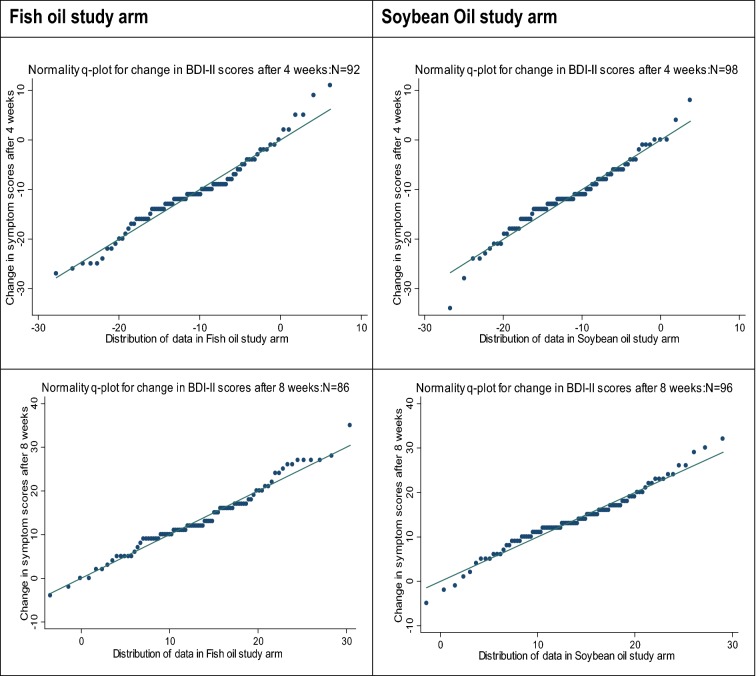
Quantile–quantile plot normality test for change in BDI-II scores by study arm The quantile–quantile plot normality statistical test for change in BDI-II depressive symptom scores conducted showed that the scores were normally distributed after 4 weeks: fish oil, *N* = 92; soybean oil, *N* = 98; and after 8 weeks: fish oil, *N* = 86; soybean oil—*N* = 96). Any variability present in the distribution of the scores in the samples was not statistically significant, based on Levene’s test of variance for sample means of the change in scores after 4 weeks (*F* (1188) = 0.79, *p *= 0.37; medians (F (1180) = 0.80, *p *= 0.37) and after 8 weeks (*F* (1180) = 1.96 *p *= 0.16); medians (*F* (1180) = 1.45, *p *= 0.23) and standard deviation test of variance (*p *= 0.24). Variability in errors across observations was controlled for through robust analysis method. Hence, the change in BDI-II depressive symptom scores met the regression analysis assumptions of normality

Based on the Shapiro–Wilk statistical test of normality, the change in BDI-II depressive symptom scores was normally distributed at week four after intervention (fish oil: *w *= 0.97, *p *= 0.07; soybean oil: *w *= 0.98, *p *= 0.13) and week eight after intervention (fish oil: *w *= 0.98, *p *= 0.35; soybean oil: *w *= 0.98, *p *= 0.24). Further statistical test for normality with normal quantile plot (q-plot) also confirmed that the change in baseline scores was normally distributed within each group at 4 weeks and after 8 weeks (Fig. 2).The change in BDI-II depressive symptom scores was normally distributed 4 weeks after intervention (fish oil: *w *= 0.97, *p *= 0.07; soybean oil: *w *= 0.98, *p *= 0.13) and 8 weeks after intervention (fish oil: *w *= 0.98, *p *= 0.35; soybean oil: *w *= 0.98, *p *= 0.24). Any variability present in the distribution of the scores in the samples was not statistically significant, based on Levene’s test of variance at 4 weeks for sample means (*F* (1188) = 0.79, *p *= 0.37) and medians (*F* (1180) = 0.80, *p *= 0.37). The variability in the distribution of data was also not statistically significant at week eight for sample means, (*F* (1180) = 1.96 *p *= 0.16), medians (*F* (1180) = 1.45, *p *= 0.23) and standard deviation test of variance (*p *= 0.24). Any difference in variability in errors across observations was controlled through robust analysis method. These statistical tests proved that the primary outcome data of the study, change in BDI-II depressive symptom scores, met the regression analysis assumptions of normality.

#### Change in BDI-II scores before adjusting for baseline covariates

At the end of the trial, at week 8, most participants in both arms had mild depressive symptoms (Fish oil = 82/86 (95.3%), Soybean oil = 94/96 (97.9%)) with a minimum BDI-II score of 19 compared to 14 at baseline. The difference in the proportions of participants with different BDI-II depressive symptom levels after 8 weeks of intervention was not significantly different between the two intervention arms (X^2^ = 3.1, d.f = 2, *p* value = 0.21).

Table 3 shows the mean change and mean difference in change in BDI-II scores before adjusting for baseline covariates at 4 and 8 weeks post intervention. After 4 weeks, fish oil intervention group had a mean (SD) change in BDI-II depressive symptom scores of − 10.8 (± 7.3) with a 95% confidence interval (CI) of − 12.3 to − 9.3 scores. The mean (SD) change in scores after the 4 weeks for the soybean oil control group was − 11.5 (± 6.5) with a 95% CI of − 12.3 to − 10.2 scores. At the end of the 8-week study period, participants in the fish oil intervention group had a mean (SD) change in BDI-II depressive symptom scores of − 13.3 (± 7.4) with a 95% CI of − 14.9 to − 12.0 scores. The mean (SD) change in scores among soybean oil control group was − 13.9 (± 6.5) with a 95% CI of − 15.3 to − 12.6 scores.

**Table 3:** Change in BDI-II scores after intervention

Intervention period	Intervention group	Change in BDI-II scores (Mean (SD, 95% CI)	Difference between groups in change in BDI-II scores (Mean (SE, 95% CI)	Statistical test		
				*t*	df	*p*
After 4 weeks	Fish oil (*N *= 92)	− 10.8 (− 12.3 to − 9.3)	0.7 (1.0, − 1.3 to 2.7)	0.69	188	0.49
	Soybean oil (*N *= 98)	− 11.5 (− 12.3 to − 10.2)				
After 8 weeks	Fish oil (*N *= 86)	− 13.3 (− 14.9 to − 12.0)	0.6 (1.0, − 1.5 to 2.6)	0.55	180	0.58
	Soybean oil (*N *= 96)	− 13.9 (− 15.3 to − 12.6)				

*SD* standard deviation

The mean difference in the change in BDI-II scores between the two intervention groups was 0.7 (Standard Error = 1.0) with a 95% CI 1.3 to 2.7 after 4 weeks and 0.6 (Standard Error = 1.0) with a 95% CI 1.5 to 2.6 after 8 weeks of intervention. These differences in change in BDI-II scores were, however, not significantly different (week 4: *p *= 0.49; week 8: *p *= 0.58) as seen in Table 3. By fitting simple linear regression model, the intervention could only explain 0.25% of the variability in change in depressive symptom scores at week four (R^2^ = 0.0025) and 0.07% (R^2^ = 0.0017) at week eight. The intervention alone was not statistically significant in explaining the variability in change in BDI-II scores at week four 4 (*F* (1188) = 0.47, *p *= 0.49) and week eight (*F* (1180) = 0.31; *p *= 0.58).

**Fig. 3:**
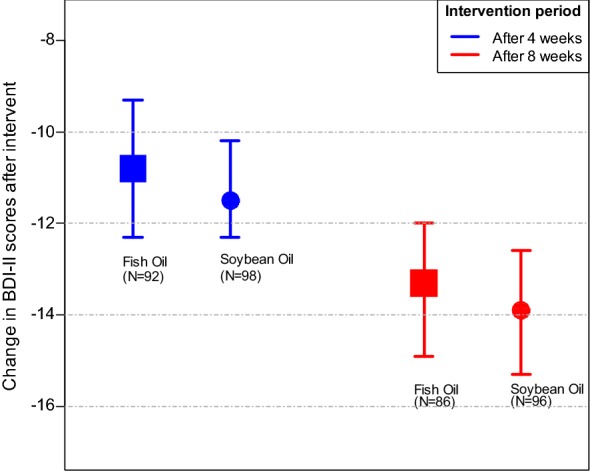
95% confidence interval bars for change in BDI-II scores by study arm and period. The calculated 95% confidence intervals (CI) for the mean difference in the change in BDI-II scores between the two intervention groups overlapped substantially after 4 weeks (Fish oil (*N *= 92). 95% CI − 12.3 to − 9.3; soybean oil (*N *= 98), 95% CI − 12.3 to − 10.2) and after 8 weeks (Fish oil (*N *= 86), 95% CI − 14.9 to − 12.0; soybean oil (*N *= 96), 95% CI − 15.3 to − 12.6). This overlap suggested that there was no significant difference in intervention effect on depressive symptoms between the two study groups

The calculated 95% CI for the two groups overlapped substantially at week four and week eight when the median scores were compared (Fig. 3), suggesting that there was no significant difference in intervention effect on depressive symptoms between the two study groups.

#### Analysis of change in BDI-II scores between groups

All baseline attributes held constant, the change in BDI-II scores at week four were 0.16 (95% CI − 1.48 to 1.81) and at week eight, 1.01 scores (95% CI (− 0.58 to 2.60) times higher in fish oil intervention group than in soybean oil control group (Table 4). This intervention effect was, however, not statistically significant at both week 4 (*p *= 0.84) and week 8 (*p *= 0.21). The baseline characteristics in the ANCOVA model explained 48.8% (R^2^ = 0.488) and 56.9% (R^2^ = 0.569) of the variance in changes in BDI-II depressive symptoms scores at week 4 and week 8, respectively.

**Table 4:** ANCOVA analysis of mean change in BDI-II scores by baseline characteristics and intervention duration

Baseline characteristics	Duration of intervention			
	Week 4		Week 8	
	Regression coefficient (95% CI)	*p* value	Regression coefficient (95% CI)	*p* value
Intervention group	0.40 (− 1.25 to 2.05)	0.63	1.00 (− 0.58 to 2.60)	0.21
Baseline BDI-II scores	− 0.79 (− 0.94 to − 0.64)	0.00*	− 0.87 (− 1.02 to − 0.73)	0.00*
Age (single years)	0.02 (− 0.16 to 0.21)	0.82	0.11 (− 0.06 to 0.28)	0.21
Gestational age (months)	0.03 (− 0.21 to 0.26)	0.83	0.04 (− 0.17 to 0.25)	0.69
CD4 cell count (cells/mm3)	0.01 (− 0.00 to 0.01)	0.12	− 0.001 (− 0.01 to 0.01)	0.62
Employment	− 0.85 (− 2.79 to 1.09)	0.39	− 58 (− 2.19 to 1.05)	0.47
Knew HIV status when pregnant	− 1.54 (− 3.33 to 0.26)	0.09	− 0.83 (− 2.71 to 1.02)	0.37
HIV status disclosure	− 1.16 (− 3.09 to 0.76)	0.24	− 0.49 (− 2.22 to 1.22)	0.57
Marital status	1.03 (− 1.02 to 3.08)	0.32	0.84 (− 0.98 to 2.66)	0.36
Education level	− 0.51 (− -2.37 to 1.35)	0.59	− 0.40 (− 2.09 to 1.29)	0.64
Parity	− 0.99 (− 2.06 to 1.86)	0.92	− 1.92 (− 4.10 to 0.26)	0.08
Stressful life event	− 0.19 (− 1.99 to 1.62)	0.84	1.01 (− 0.48 to 2.59)	0.18
PMTCT *m2* *m* meetings	2.04 (0.37 to 3.70)	0.02*	1.09 (− 0.44 to 2.65)	0.16
MUAC (cm)	0.03 (− 0.34 to 0.39)	0.87	− 0.38 (− 0.35 to 0.27)	0.81
Dietary omega-3	− 2.98 (− 5.32 to − 0.64)	0.01*	− 0.32 (− 2.25 to 1.97)	0.89
Dietary vitamin C	− 0.85 (− 2.70 to 0.99)	0.36	0.94 (− 0.97 to 2.85)	0.33
Dietary vitamin B1	− 3.26 (− 5.21 to − 1.31)	0.001*	− 1.41 (− 3.65 to 0.83)	0.22
Dietary vitamin B6	2.27 (− 0.37 to 4.91)	0.09	2.24 (− 0.04 to 4.51)	0.05*
Dietary vitamin B12	0.07 (− 2.42 to 2.57)	0.95	− 0.63 (− 2.80 to 1.56)	0.57
Dietary folate	0.62 (− 2.02 to 3.27)	0.64	1.44 (− 1.29 to 4.18)	0.30
Dietary vitamin E	− 0.59 (− 3.27 to 2.09)	0.66	− 1.29 (− 3.19 to 0.62)	0.18
Dietary zinc	8.67 (− 4.49 to 21.8)	0.19	5.39 (0.64 to 10.14)	0.03*
Dietary selenium	1.23 (− 1.20 to 3.66)	0.32	1.84 (− 0.25 to 3.94)	0.08
Dietary calcium	1.23 (− 0.72 to 3.27)	0.21	− 1.64 (− 3.60 to 032)	0.10

^*^Baseline variables that were significantly associated with the change in BDI-II depressive symptom scores at week 4 and week 8

At week 4, the change in BDI-II depressive symptom scores was significantly associated with baseline BDI-II scores, − 0.77 (95% CI − 0.94 to − 0.61, *p *= 0.000), dietary omega-3 intake − 3.03 (95% CI − 5.41 to − 0.66, *p *= 0.012), dietary vitamin B1 intake − 3.46 (95% CI − 5.46 to − 1.48, *p *= 0.001), attendance in PMTCT m2 m support group meetings 2.06 (95% CI 0.38 to 3.74, *p *= 0.02), vitamin B6 2.84 (95% CI 0.24 to 5.42, *p *= 0.03) and zinc-2.60 (95% CI − 4.42 to − 0.78, *p *= 0.01). The presence of interaction between baseline BDI-II depressive symptom scores and these variables that were significantly associated with change in BDI-II scores was tested. There was no interaction between the baseline BDI-II scores and any of the variables, suggesting that each variable influenced the change in BDI-II scores independently.

At the end of the study at week 8, change in BDI-II depressive symptom scores was significantly associated with baseline BDI-II symptom scores − 0.87 (95% CI − 1.02 to − 0.72; *p *= 0.000) as seen on Table 4. The change in the depressive symptom scores was slightly more than two times higher, 2.24 (95% CI − 0.04 to 4.51; *p *= 0.05) among participants who met the estimated average requirements for Vitamin B6 dietary intake at baseline. The change was also more than five times higher, 5.39 (95% CI 0.63 to 10.14, *p *= 0.03) among those participants who met the estimated average requirement for their dietary zinc intake at baseline, assuming that all other variables were held constant in the ANCOVA model. Dietary iron intake was, however, omitted in the analysis because all participants in both study groups (Fish oil: 109/109 and soybean oil: 107/107) had their intake levels below the recommended EAR values. The presence of interaction between baseline BDI-II depressive symptom scores and dietary zinc and vitamin B6 intake was tested. There was no interaction between the baseline BDI-II scores and any of the two variables and each variable influenced the change in the BDI-II scores independently.

#### Adverse events

Both known and unknown possible adverse events were closely monitored throughout the 8-week study period for each participant by phone or face-to-face individual interviews and observations. There were no serious adverse events reported by participants from either of the groups. However, some participants reported that they occasionally experienced unpleasant feelings as shown on Table 5.

**Table 5:** Participants reporting unpleasant feelings by study arm and period

Other adverse events (not serious) experienced	Soybean oil	Fish oil
Total reported	15/107 (14.02%)	24/109 (22.02%)
Occasionally nauseated with fishy-after-taste	5/107 (4.67%)	10/109 (9.17%)
Occasional vomiting in the morning after taking soft gel	3/107 (2.8%)	6/109 (5.5%)
Occasional heartburn	5/107 (4.67%)	3/109 (2.75%)
Occasional bloated stomach	0/107 (0%)	2/109 (1.83%)
Occasional loose stool	1/107 (0.93%)	0/109 (0%)
Occasional itchy skin	1/107 (0.93%)	2/109 (1.83%)
Nose bleeding once	0/107 (0%)	1/109 (0.92%)

## Discussion

In this study, we tested the hypothesis that there is no difference in the magnitude of change in BDI-II scores between HIV-seropositive pregnant women with depressive symptoms taking fish oil omega-3 EPA-rich supplements and the control group taking soybean oil soft gels. We had hypothesized to detect as statistically significant at 5% level (α = 0.05) a true difference of at least 4 BDI-II scores, equivalent to a 20% change, between the intervention and control group. All participants were randomized at same level in terms of their demographic, socio-economic, dietary, depressive symptoms and other health-related attributes. Any imbalance that might have occurred was therefore due to chance alone. At the end of the 8-week trial period, each group experienced a 20% change (4 units) in BDI-II scores. The differences in reduction in BDI-II scores were 0.7 and 0.6 times more in the fish oil intervention group than in the soybean oil control group at week four and week eight, respectively. These differences were not statistically significant. The fish oil intervention alone could only explain 0.25% and 0.07% of the variability at week four and week eight, respectively. The change in BDI-II depressive symptom scores was only 0.14 (95% CI − 1.51 to 1.78) times higher at week four and 1.01 scores (95% CI (− 0.58 to 2.60) times higher at week eight in fish oil intervention group than in soybean oil control group. The statistical change in the BDI-II scores could not be explained by either fish oil or soybean oil alone.

Our study demonstrated that there is no difference in the magnitude of change in BDI-II scores between HIV–seropositive pregnant women with depressive symptoms taking fish oil omega-3 EPA-rich supplements with a daily dosage of 3.17 grams (EPA = 2.15 grams; DHA = 1.02 grams) and a control group taking soybean oil of a daily dosage of saturated fatty acids (0.53 grams), monounsaturated fatty acids (0.89 grams) and polyunsaturated fatty acids (0.985 grams) with traces of EPA (0.34 grams). Based on this, we conclude in this study that the fish oil omega-3 is not effective in reduction of depressive symptoms among HIV-infected pregnant women with mild, moderate and severe depression symptoms. The fish oil omega-3 supplements were, however, well tolerated, without serious adverse side effects among HIV-infected pregnant women.

Several possible explanations could have contributed to the observed lack of a statistically significant difference in the change in BDI-II scores between the fish oil intervention group and the soybean oil control group. First, in this study, participants had mild, moderate and severe depressive symptoms at baseline while previous studies had only participants with major depressive symptoms. Second, the previous studies on omega-3 supplementation were conducted among pregnant women without HIV infection. Even though the prevalence of depression in HIV-infected pregnant women has been documented before [11, 13, 53, 54], change in depressive symptoms scores following omega-3 EPA-rich fatty acid supplementation had not been previously demonstrated in this population group. Yet, in HIV-infected pregnant women, the omega-3 EPA and DHA fatty acid absorption may be impaired [14, 55] due to reduced activity of the pancreatic lipase enzyme [56], thus contributing to low response to the fish oil omega-3 fatty acid supplementation.

The third explanation for lack of a statistically significant difference in change in BDI-II scores between the groups could have been the “placebo effect”. Due to the “placebo effect”, some participants in the soybean oil control group believed that they were taking fish oil soft gels and reported a reduction in their depressive symptom scores might have contributed to the observed lack of a significant statistical difference between the intervention and control group. Both the fish oil soft gels for the intervention group and soybean oil for the control group were physically similar in shape and color. This “placebo effect” might have influenced the mean change in BDI-II depressive symptom scores of the groups, causing “regression to the mean” value, where the mean change in depressive symptom scores showed a trend towards the treatment effect [57]. “Regression to the mean” was controlled in this study through the ANCOVA analysis model, which adjusted for any extreme baseline values and variability during analysis.

The fourth possible explanations for lack of a statistically significant difference in change in BDI-II scores between the fish oil intervention group and the soybean oil control group in this study was the intensive follow-ups of participants throughout the study period which could have contributed to “Hawthorn Effect”. McCarney and colleagues demonstrated that intensive follow-up of participants resulted in a better outcome in clinical trial than minimal follow-up, and they defined “Hawthorn Effect” as increase in treatment response due to psychological stimulus of being singled out and being made to feel important [58]. Regular follow-ups of all participants in both study groups after every 2 weeks to re-supply the treatment, and regular cell-phone contacts for purposes of monitoring adherence in taking the treatment might have made the HIV-infected pregnant feel worthwhile and more positive about their pregnancy outcome. The findings on depressive symptoms revealed that the symptom of worthlessness was reported by more than 60% of participants from intervention and control group at baseline, but less than 25% of participants from each group at the end of the study. This is an indication that more than 75% of participants had their BD-II score for the symptom of worthlessness reduced at the end of the study. Although routine PMTCT-*m2m* peer education meeting attendance did not significantly statistically influence the change in BDI-II scores, its activities might have also contributed to the positive response and feeling of “important” exhibited by participants during follow-ups in both study groups. It was not possible to control for the Hawthorn Effect. Regular follow-up of participants in this study was, however, necessary to re-supply the intervention and monitor compliance in the trial.

Although there was no significant difference in baseline BDI-II scores between the intervention and control groups before randomization, participants who had higher BDI-II scores at baseline experienced less change in their depressive scores than those who had lower scores. The findings suggest that severity of depressive symptoms was a predictor of the magnitude of change in BDI-II depressive symptoms scores rather than the fish oil omega-3 intervention. These findings on severity of baseline depressive symptom scores influencing change in the scores after intervention support the observation made by Kilt and colleagues that response to depression treatment decreased with increasing baseline symptom severity [59]. These research findings highlight the need for routine screening for depressive symptoms to guide timely identification and management of depressive symptoms in HIV-infected pregnant women.

### Strengths and weaknesses of the study

To our knowledge, this was the first study to explore the effect of fish oil omega-3 fatty acids on depression among HIV-infected pregnant women. It examined change in individual pregnant women’s BDI-II depressive symptom scores before and after the intervention with fish oil omega-3 EPA-rich supplement. Most potential confounding demographic, socio-economic and health-related factors were controlled for during the study design by the randomization. The ANCOVA analysis model used in analyzing the study outcome further controlled for any imbalance due to baseline variations. Other baseline covariates which were most likely to change over the intervention period such as BDI-II depressive symptom scores, CD4 cell count levels, frequency of interaction with PMTCT peer educators, occurrence of stressful life events and omega-3 fatty acid levels in the body were controlled for in the ANCOVA analysis model.

The other strength of this study was the use of BDI-II tool which contains depressive symptoms exhibited in both HIV and pregnancy conditions. Our statistical reliability test for the BDI-II scale in assessing depressive symptoms in HIV-infected pregnant women confirmed that the tool was reliable. The types of depressive symptoms exhibited in pregnancy and HIV infection that were reported by a sub-sample of HIV-infected pregnant women and health workers prior to baseline data collection for the main study also confirmed that the local understanding of depressive symptoms matched with the symptoms listed on the BDI-II tool. Furthermore, the use of BDI-II tool in screening for depressive symptoms had been previously validated in other studies among adults, pregnant women and HIV-infected individuals and in the African context [60–62].

Our study findings may not be generalized to other populations who are neither pregnant nor HIV seropositive. This is for two reasons. First, participants were depressed partly because of their HIV condition, pregnancy or a combination of both HIV and pregnancy. However, the BDI-II depressive symptom screening tool used in this study comprises of a comprehensive list of symptoms that are exhibited in both pregnancy and HIV infection. Second, pregnancy and HIV infection on their own are also associated with nutrient depletion and increased nutrient demand. This implies that the recommended EARs for pregnancy alone are not adequate for pregnancy and HIV infection conditions. This research, however, contributes to the ongoing debate on the effect of fish oil omega-3 fatty acids in reduction of depressive symptoms among pregnant women in the context of HIV infection. Further larger studies with a higher dose of fish oil supplements without soybean oil for control groups should be explored, particularly among the severely depressed women from this vulnerable population in future studies. Such studies in future should also consider controlling for the “Hawthorn effect” throughout the study period.
